# Early Life Stress Promotes Heroin Seeking But Does Not Alter the Excitability of Insular Pyramidal Cells Targeting the Nucleus Accumbens

**DOI:** 10.3389/fnbeh.2021.777826

**Published:** 2021-12-07

**Authors:** Jonna M. Leyrer-Jackson, Paula F. Overby, Erin K. Nagy, M. Foster Olive

**Affiliations:** Department of Psychology, Arizona State University, Tempe, AZ, United States

**Keywords:** limited bedding and nesting, early life stress, heroin, electrophysiology, self-administration, anterior insula

## Abstract

A number of retrospective studies have demonstrated adverse childhood experiences are associated with increased vulnerability to substance use disorders, including opioid use disorders (OUDs). These adverse childhood experiences, also referred to as early life stress (ELS), can be modeled in laboratory animals by various paradigms including limited bedding and nesting (LBN) procedures. Studies using rodent models of ELS have been shown to recapitulate various aspects of OUDs, including relapse propensity and perseverance of drug-seeking behavior. In the current study, we utilized the LBN paradigm to explore potential effects on heroin self-administration, extinction, and relapse-like behaviors in male and female rats. We also utilized *in vitro* whole-cell electrophysiology to examine the effects of LBN and repeated heroin administration on the excitability of pyramidal neurons in the anterior insular cortex (AIC) projecting to the nucleus accumbens core (NAc), as recent studies suggest that this circuit may mediate various aspects of OUDs and may be compromised as a result of either ELS or OUDs. We observed that compared to control animals, rats exposed to LBN conditions during postnatal days 2–9 showed increased breakpoints for heroin self-administration under a progressive ratio schedule of reinforcement, impaired extinction of heroin-seeking behavior, and increased reinstatement of heroin-seeking behavior induced by heroin-associated cues. No effect of LBN rearing conditions were observed on the acquisition and maintenance of heroin self-administration, and no sex differences in heroin intake were observed. LBN and control reared animals showed no differences in the excitability of AIC-NAc pyramidal neurons, but animals treated with repeated heroin showed decreased excitability of these neurons through a significant increase in rheobase and reduction in action potentials induced by depolarizing currents. Together, these results suggest that ELS exposure produces exacerbations of heroin seeking behavior without parallel effects on AIC-NAc excitability, although heroin itself reduces the excitability of these neurons.

## Introduction

Opiate abuse and addiction pose a substantial financial economic burden, exceeding over one trillion dollars in the United States in 2017 (Florence et al., 2021). The epidemic has been fueled by multiple issues including over-prescription of opiate analgesics, miseducation of physicians and patients about addiction liability, and the increased availability of low-cost heroin. In 2020, opioid-related overdoses in the United States surpassed 93,000, representing a 30% increase over the previous year (Chappell, 2021), an effect likely perpetuated by the coronavirus pandemic (Seervai, 2021). Opioid use disorders (OUDs) are, in large part, perpetuated by negative affect, where continuance and/or resumption of drug intake is driven by the alleviation of severe negative affective states that occur during opiate withdrawal (i.e., dysphoria, depression, and anhedonia) (Koob, 2020; Welsch et al., 2020). Environmental influences on opiate addiction, including early life stress (ELS) and other psychosocial stressors such as discrimination and socioeconomic impoverishment, increase vulnerability toward the development of OUDs (Lupien, 2014; Scarna, 2020). In fact, individuals with >5 instances of adverse childhood experiences are 7–10 times more likely to report illicit drug use problems, including intravenous opiate use and dependence, than individuals without a history of ELS (Dube et al., 2003). Therapies for patients with OUDs have largely focused on pharmacological maintenance strategies which aide in minimizing opiate withdrawal, yet the existing literature strongly indicates that the use of other strategies such as psychosocial interventions (e.g., counseling, family therapy, community reinforcement), when used in combination of pharmacological approaches, are particularly effective at reducing relapse (Wild et al., 2021).

In humans, ELS includes a myriad of experiences including physical, emotional or sexual abuse, neglect, and instability of maternal care. Such events have been shown to increase risk of developing impairments in coping skills, affective processing, and impulse control, all of which are characteristics of individuals with OUDs (Pechtel and Pizzagalli, 2011; Lovallo, 2013). Such manifestations are often accompanied with abnormal functional connectivity and brain development. In rodents, ELS can influence the initiation and maintenance of drug self-administration and relapse-related behaviors (Duffing et al., 2014; Koob and Schulkin, 2019). ELS exposure in rodents is typically conducted during a critical neurodevelopmental stage, usually within the first 2 weeks of postnatal life. One commonly used ELS mimicking childhood neglect and instability of maternal care is the limited bedding and nesting (LBN) paradigm, where dams are provided insufficient material for nesting within the home cage, which results in maternal distress and dysregulated pup care (Gilles et al., 1996; Molet et al., 2014; Walker et al., 2017). Rodents exposed to the LBN paradigm often show increased vulnerability to drug-seeking, alterations in reward- and stress- related circuits as well as increased negative affect (Moffett et al., 2007; Walker et al., 2019). In fact, LBN procedures produce increased locomotor sensitization to opioids, increased withdrawal severity, as well as increased magnitude of cue- and drug-primed reinstatement of opioid-seeking behavior (Kalinichev et al., 2001; Vazquez et al., 2006; Michaels and Holtzman, 2008). However, it remains unknown how ELS can alter single cell physiology of neuronal networks that gives rise to these behavioral phenotypes.

The insular cortex shares reciprocal connections with various brain regions including limbic regions associated with reward, such as the nucleus accumbens (NAc). Functional neuroanatomical studies have demonstrated that the anterior insular cortex (AIC) mediates a number of behaviors including cognitive, emotional, integrative and social functions, including attention, decision-making, and incentive and risk assessment. In rodents, lesions of the AIC or pharmacological modulations of this region alter cocaine intake and cocaine-seeking behaviors (Arguello et al., 2017), conditioned opiate reward and aversion (Wu et al., 2014; Wang et al., 2016), heroin self-administration (Joshi et al., 2020) and nicotine self-administration (Scott and Hiroi, 2011), confirming its role in addiction-related behaviors. However, precise neuroanatomical studies examining circuits involving the AIC that mediate these behaviors, especially in regards to heroin seeking, are sparse. Recent studies have demonstrated that excitatory projections from the AIC to subcortical regions such as the amygdala and NAc mediate both alcohol consumption and cue-induced reinstatement of morphine conditioned reward (Jaramillo et al., 2018; Zhang et al., 2019; Haaranen et al., 2020), however studies have yet to be conducted examining effects of LBN and/or ELS on single-cell physiology in the AIC.

In the current study we aimed to explore how ELS, as modeled through the LBN paradigm, alters heroin seeking through intravenous self-administration (IVSA), extinction of heroin-seeking, as well as reinstatement elicited by exposure to heroin-paired cues. Further, we also explored the effects of LBN on the single-cell physiology and excitability of AIC neurons projecting to the NAc. Additionally, we examined the effects of experimenter delivered heroin to LBN reared and control animals to determine if heroin further altered single cell physiology and excitability of these neurons, as such alterations may give rise to behavioral alterations such as heroin seeking and relapse.

## Materials and Methods

### Animals

A total of 39 Long-Evans rats were used in the current study. Four pregnant dams were ordered at gestation day 10 from Charles River Labs (Wilmington, MA, United States). All dams were kept in a temperature-controlled vivarium (22–24°C) at Arizona State University on a standard reverse light-dark cycle (12 hr light/12 hr dark). Rats were single housed in conventional polycarbonate cages until pups were weaned. All animals were given standard rodent chow and water *ad libitum* for the duration of the study. All experiments conducted were approved by the Institutional Animal Care and Use Committee at Arizona State University.

### Limited Bedding and Nesting

A total of 4 litters were used in the current study. At PND2, the sex of each pup was determined *via* anogenital distance. To minimize potential genetic influences, pups from all litters were randomly assigned to one of two conditions (LBN or control), and sex was kept at a 1:1 ratio. To minimize potential litter influences, dams assigned to the LBN group were placed with their assigned pups in a standard cage containing a wire mesh floor designed to maintained animals ∼2.5 cm above the cage floor, which contained a thin layer of standard bedding. However, due to some pups becoming entrapped in the mesh during the 1st day of LBN procedures (PND 2), wire meshes were removed from the cages and dams assigned to the LBN condition were provided only with ∼100 ml of standard bedding and a single folded paper towel to use as nesting material (Walker et al., 2017) for the remainder of LBN procedures (PND3-9). Control dams and pups were housed in cages containing standard amounts of bedding and nesting material. Dams and pups were returned to normal housing during PND10–21. On PND21, all pups were weaned and same sex pair housed until PND70, where animals either underwent surgical implantation of jugular vein catheters for intravenous heroin self-administration (Experiment 1), or stereotaxic injection of retrograde AAV expressing enhanced green fluorescent protein (EGFP; Experiment 2). Following surgical procedures, all animals were single housed for the duration of the experiment. All dams were only used once to avoid influences of LBN on maternal behaviors in subsequent litters.

### Surgical Procedures

All animals used in Experiment 1 (*n* = 24; 12 males, and 12 females) were anesthetized with isoflurane (5% induction, 2–3% maintenance) vaporized in oxygen at a flow rate of 2 L/min and implanted with polyurethane catheters (BTPU-040; Instech Laboratories, Plymouth Meeting, PA) 2–2.5 cm into the right jugular vein and were tunneled subcutaneously to the posterior side of the animal and connected to an indwelling back port (Instech). Dental cement was used to adhere the catheter to the indwelling port, and the port was sutured ∼2 cm caudal from the shoulder blades using 4–0 vicryl braided sutures. Catheters were flushed daily with heparin (70 U/ml) containing timentin (66.6 mg/ml) during recovery to maintain patency and minimize postoperative infections. Meloxicam (1 mg/kg, s.c.) was administered immediately following surgery and once daily for the first 3 days of recovery to minimize discomfort. Throughout the remainder of the self-administration phase of the experiment, heparin (10 U/ml) was administered daily. Animals used in Experiment 2 (*n* = 15; 8 males, and 7 females) were anesthetized as described above underwent stereotaxic injections of the retrograde serotype AAV expressing EGFP (AAVrg-CAG-EGFP; titer >10^12^ vg/ml; Addgene #37825) into the nucleus accumbens core (NAc) at a volume of 300—400 nl and at the following coordinates in mm from bregma and skull surface: A/P: + 2.5; M/L ± 1.6; DV: −6.1. The AAV was delivered over a 5 min period and the injector was left in place for an additional 10 min to allow for diffusion. The wound was then sutured closed and triple antibiotic ointment was applied to the suture site. Meloxicam (1 mg/kg s.c.) was administered immediately following surgery and for 6 consecutive days during recovery.

### Heroin Self-Administration

**FIGURE 1:**
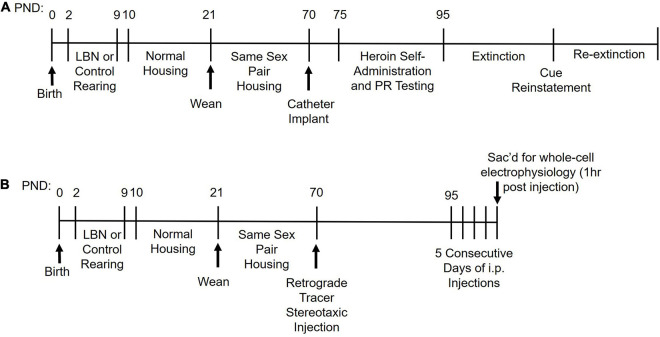
A timeline of experimental procedures for **(A)** Experiment 1 and **(B)** Experiment 2.

All self-administration procedures were conducted in Med Associates operant conditioning chambers as described elsewhere (Nagy et al., 2020). Animals were allowed to self-administer heroin (0.06 mg/kg/inf) *via* nosepoke on an FR1 reinforcement schedule in 3 hr daily sessions. A stimulus light and audible cues (2900 Hz tone and the sound of the syringe pump motor) were presented during each heroin infusion. After 15 days of self-administration, rats were subjected to progressive ratio (PR) testing where an exponential increase in the number of active nosepokes (i.e., 1, 2, 4, 6, 9, 12, 15, 20, 25, 32, etc.) was required to obtain each successive infusion. PR sessions were conducted in 3 hr daily sessions across for three consecutive days, and resulting data were averaged across days for analysis. Animals were then allowed to re-establish baseline IVSA patterns on an FR1 schedule for 2 days. Next, animals underwent extinction procedures where active nosepokes no longer produced heroin infusions or cue presentations. After reaching extinction criteria (>70% reduction in the number of active nosepoke responses as compared to the average value on the final 2 days of self-administration) animals were placed in their home cages and left undisturbed for 2 days prior to cue-induced reinstatement of heroin-seeking. For reinstatement sessions, previously paired heroin-associated cues (stimulus light, tone, and sound of the syringe pump motor) were presented upon active nosepokes. On the day following cue reinstatement testing procedures, active nosepoke responding was again extinguished using the same procedures and criteria above. A timeline of these procedures is outlined in Figure 1.

### Whole-Cell Electrophysiology

Prior to whole-cell electrophysiology, animals used for Experiment 2 were randomly assigned to receive once daily intraperitoneal (i.p.) injections of either saline or heroin (2 mg/kg) for five consecutive days. This dosage of heroin was selected given its ability to produce conditioned place preference (CPP) in rodents (Schlussman et al., 2008). One hour following the last injection, rats were asphyxiated with CO_2_, rapidly decapitated and brains were extracted. Brains were placed into ice-cold carbogen (95% O_2_/CO_2_) saturated cutting solution containing the following: 120 mmol/l NaCl, 25 mmol/l NaHCO_3_, 10 mmol/l dextrose, 3.3 mmol/l KCl, 1.23 mmol/l NaH_2_PO_4_, 1.8 mmol/l CaCl_2_, and 2.4 mmol/l MgCl_2_, osmolarity adjusted to 295 ± 5 mOsm and pH adjusted to 7.40 ± 0.03. Coronal slices (300 μm) containing the insular cortex were then prepared in ice-cold cutting buffer using a vibrating tissue slicer (Leica, VT 1000S). Slices were then transferred to a holding chamber filled with recording artificial cerebrospinal fluid (aCSF) containing in the following: 120 mmol/l NaCl, 25 mmol/l NaHCO_3_, 3.3 mmol/l KCl, 1.23 mmol/l NaH_2_PO_4_, 0.9 mmol/l CaCl_2_, 2.0 mmol/l MgCl_2_, and 10 mmol/l dextrose, osmolarity adjusted to 295 ± 5 mOsm and pH adjusted to 7.40 ± 0.03. In the holding chamber, the recording solution was continuously saturated with carbogen and incubated at 34°C for 45 min, then allowed to cool at room temperature before being transferred to the recording chamber. Once transferred to the recording chamber, slices were perfused continuously with carbogen saturated recording solution. Pyramidal cells within the insular cortex expressing EGFP were visualized using an Olympus BX51WI microscope with infrared DIC microscopy and equipped with a blue collimated LED (ThorLabs). Whole-cell recordings were made from the soma of pyramidal neurons expressing EGFP using recording pipettes (7–15 mΩ) made from thin-walled capillary tubes containing an intracellular solution containing the following: 135 mmol/l K-gluconate, 12 mmol/l NaCl, 1 mmol/l K-EGTA, 10 mmol/l HEPES, 2 mmol/l Mg-ATP, and 0.38 mmol/l tris-GTP. Osmolarity was adjusted to 285 ± 5 mOsm and pH adjusted to 7.30 ± 0.01. Upon establishing a giga-seal, the cell membrane was ruptured and cells were allowed to equilibrate for 5 min until a steady state resting membrane potential was reached. In voltage clamp mode, a 10 mV square pulse was delivered *via* the recording pipette and 100 capacitive transients were recorded and averaged to determine membrane capacitance/resistance and series resistance. Recordings were then switched to current clamp mode, where rheobase and input-output (I-O) curves were assessed to determine the amount of injection current required to elicit action potential spiking. A total of 15 depolarizing current pulses (150 ms duration, stepped from −35 pA to 175 pA in 15 pA increments) were used. Action potential (AP) spike threshold, amplitude, rise time, and decay time were also measured, as were after-hyperpolarization (AHP) amplitude and duration. Each of these protocols have been described in detail in our prior studies (Leyrer-Jackson et al., 2020, 2021). Successful recordings from a total of 70 labeled cells (*n* = 17–18 cells/group).

### Data Analysis

Self-administration data were analyzed utilizing a two-way ANOVA with session and rearing condition (LBN and control) as considered factors. Extinction and re-extinction data were analyzed using a survival analysis, where LBN and control reared animals were compared. Cue-induced reinstatement data were analyzed utilizing a two-way ANOVA with session (extinction and cue-induced reinstatement) and rearing condition (LBN and control) as considered factors. Electrophysiological data were analyzed using a two-way ANOVA, where group (LBN and control) and treatment (saline and heroin) were considered factors. Bonferroni-corrected *post hoc t*-tests were conducted where appropriate and *p*-values <0.05 were considered statistically significant. The number of *post hoc* comparisons for all two-way ANOVAs was six. Additionally, unpaired *t*-tests were used to assess changes in rheobase, action potential amplitude, action potential rise time and action potential decay time between treatment groups (saline and heroin). Lastly, a two-way ANOVA with treatment (heroin and saline) and input current amplitude as factors was used to assess changes in excitability. Bonferroni’s *post hoc* comparisons were utilized to examine changes in the number of action potentials elicited at each current input. Statistical tests were performed in GraphPad Prism 8.0, and *p* < 0.05 was considered statistically significant. Values are represented as mean ± standard error of the mean (SEM).

## Results

### Experiment 1: The Effects of Limited Bedding and Nesting on Heroin Self-Administration, Extinction and Reinstatement

#### Male and Female Rats Show Similar Acquisition Rates of Heroin Intravenous Self-Administration and Total Heroin Intake

**FIGURE 2:**
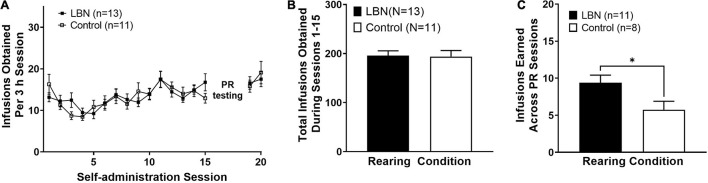
Heroin self-administration does not differ across rearing condition. Similar rates of acquisition of heroin IVSA **(A)** and total heroin intake between rearing conditions **(B)**. LBN animals showed higher breakpoints for heroin (total infusions earned, averaged across PR sessions) **(C)**. * indicates *p* < 0.05 between rearing conditions.

First, we examined acquisition of heroin IVSA across male and female animals in both the control and LBN groups. A two-way ANOVA, where session and rearing condition were considered factors, revealed a significant effect of session (*F*_16,336_ = 5.4; *p* < 0.0001) but not rearing condition (*F*_3,336_ = 0.4; *p* > 0.05) and no interaction was observed (*F*_48,336_ = 1.0; *p* > 0.05). A Bonferroni’s *post hoc* analysis revealed no differences between rearing condition at any session (Figure 2A). Additionally, an two-way ANOVA, where sex and rearing condition were considered factors, revealed no effect of sex (*F*_1,20_ = 0.9; *p* > 0.05), rearing condition (*F*_1,20_ = 0.001; *p* > 0.05) or interaction (*F*_1,20_ = 0.1; *p* > 0.05) in the total number of infusions earned across self-administration sessions 1–15. An unpaired *t*-test revealed that LBN and control animals did not differ in the total number of infusions earned across self-administration sessions 1–15 (*t*_22_ = 0.1; *p* > 0.05; Figure 2B). The current study was slightly underpowered to detect sex differences, however, no trends in sex differences were observed in any analysis examining sex. Thus, we have collapsed by sex for analysis between control and LBN groups. We then compared the number of infusions earned across the 3 PR sessions. An unpaired *t*-test revealed that LBN animals earned significantly more infusions than those reared in the control condition (*t*_18_ = 2.3; *p* < 0.05; Figure 2C). Thus, under PR conditions, animals exposed to LBN rearing showed higher breakpoints for heroin (increased number of infusions earned) as compared to control animals, suggesting increased motivation for heroin. It is of note that four animals were removed from PR analysis. One animal was removed from the remainder of the study due to unstable responding while the other three were moved directly into extinction due to patency issues.

#### Limited Bedding and Nesting Reared Animals Show Impaired Extinction and Enhanced Reinstatement Relative to Control Reared Animals

**FIGURE 3:**
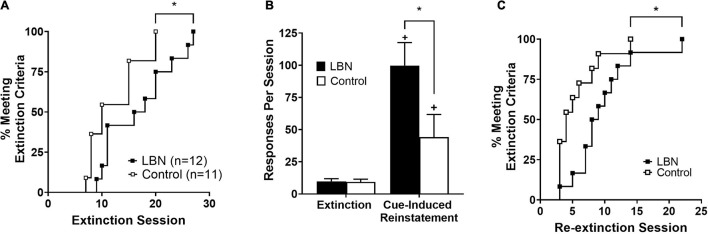
LBN reared animals show impaired extinction and robust cue-induced reinstatement relative to control reared animals. **(A)** Survival analyses showed impaired extinction of heroin-seeking in LBN animals relative to control (*p < 0.05 versus control). **(B)** Both LBN and control reared animals display cue-induced reinstatement (+ *p* < 0.05 vs. extinction values). LBN reared animals exhibited more robust cue-induced reinstatement relative to control reared animals (*p < 0.05). **(C)** Survival analyses showed impaired re-extinction of heroin-seeking in LBN animals relative to control (*p < 0.05 vs. control).

Survival analyses showed impaired extinction of heroin-seeking in LBN animals relative to control (chi-square value = 4.1; *p* < 0.05; Figure 3A). Following the first series of extinction sessions, animals were placed into cue-induced reinstatement procedures. A two-way ANOVA analyzing cue-induced reinstatement, where session (extinction and cue-induced reinstatement) and rearing condition (LBN and control) were considered factors, revealed significant effect of session (*F*_1,40_ = 5.0; *p* < 0.05), rearing condition (*F*_1,40_ = 5.2; *p* < 0.05) as well as a significant interaction (*F*_1,40_ = 25.7; *p* < 0.05) (Figure 3B). Following cue-induced reinstatement procedures, animals were subjected to the same extinction procedures, and a survival analyses showed impaired re-extinction of heroin-seeking in LBN relative to control animals (chi-square value = 3.8; *p* < 0.05; Figure 3C).

### Experiment 2: Experimenter-Administered Heroin but Not Limited Bedding and Nesting Rearing Conditions Reduces the Excitability of Anterior Insular Cortex Neurons Projecting to the Nucleus Accumbens Core

**FIGURE 4:**
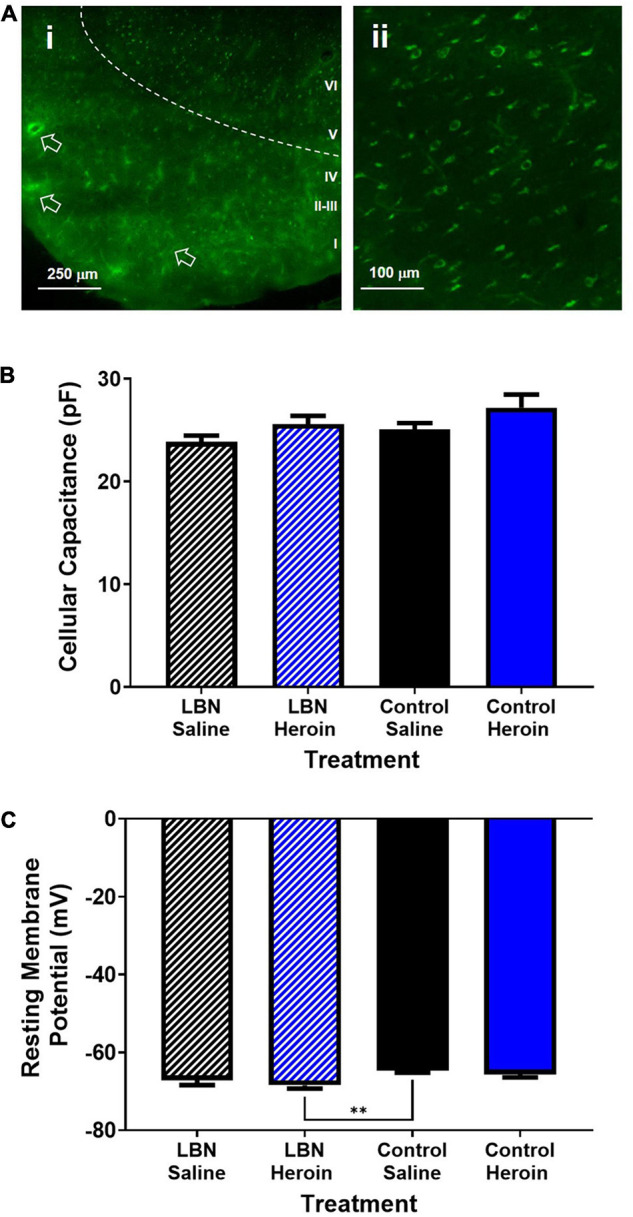
Electrophysiological characteristics of AIC-NAc projection neurons. **(A)** Low magnification image of brain slice containing retrogradely labeled AIC-NAc neurons (i). Deeper (layer V) regions of the cortex display predominant labeling (ii). Arrows indicate fluorescence in vasculature due to unperfused brain tissue. Cellular capacitance did not differ across experimental groups **(B)**. Control reared animals treated with saline had a significantly lower resting membrane potential relative to LBN reared animals treated with saline (**p < 0.01) **(C)**. However, no differences between heroin treated animals or saline treated animals was observed.

All recordings were conducted from layer V pyramidal cells within the insula that expressed EGFP from retrograde viral labeling to the core, indicating that all recorded cells had axonal projections targeting the NAc. Representative labeling of AIC-NAc projecting cells can be seen in Figure 4A. Overall, AIC-NAc projection neurons had an average cellular capacitance of 25.6 ± 0.5 pA, resting membrane potential of −66.4 ± 0.4 mV and spiking threshold of −41.5 ± 1.1 mV. A two-way ANOVA, where rearing condition and drug were considered factors, revealed a significant effect of drug (*F*_1,61_ = 4.3; *p* < 0.05) but not treatment (*F*_1,61_ = 2.2; *p* < 0.05) or interaction (*F*_1,61_ = 0.04; *p* < 0.05) between drug and treatment on cellular capacitance. A *post hoc* Bonferroni comparison revealed no significant differences between groups (*p* > 0.05; Figure 4B). A two-way ANOVA, where rearing condition and drug were considered factors, revealed a significant effect of rearing condition (*F*_1,61_ = 9.8; *p* < 0.01; Figure 4C). A Bonferroni’s *post hoc* revealed a significant difference between control reared saline treated animals and LBN reared heroin-treated animals (*t*_61_ = 3.3; *p* < 0.01; Figure 4C). Interestingly, for all other measurements described below, we found no significant differences between LBN and control reared Heroin or Saline treated animals. Thus, for all measurements described below, we have grouped Heroin animals (LBN + Control) as well as Saline animals (LBN + Control) for all analyses.

#### Repeated Heroin Administration Reduces Excitability of Anterior Insular Cortex-Nucleus Accumbens Core Projection Neurons in Both Control and Limited Bedding and Nesting Reared Animals

**FIGURE 5:**
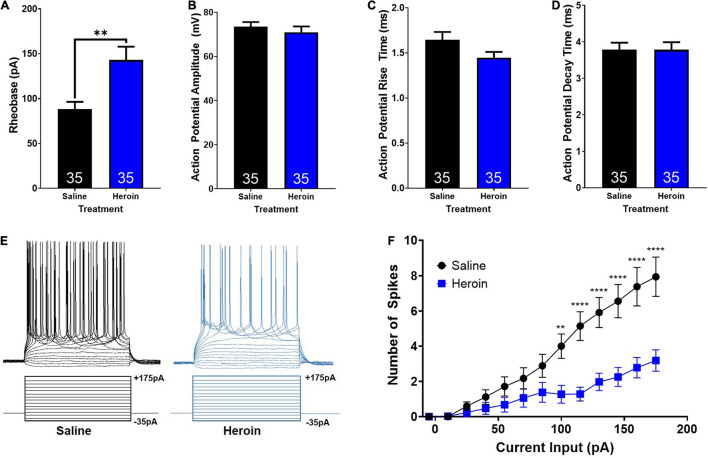
Heroin-induced changes in cellular excitability of AIC-NAc projecting neurons. **(A)** Heroin exposure significantly increases the rheobase of AIC-NAc projecting neurons relative to saline (** indicates *p* < 0.05). Heroin exposure does not alter action potential amplitude **(B)**, rise time **(C)** or decay time **(D)** relative to saline. **(E)** Example traces of action potentials (top) elicited by stepped current injections (bottom). **(F)** Repeated administration of heroin reduces the number of spikes evoked at higher current injections, relative to control (**p < 0.01; ****p < 0.0001).

First, we examined heroin-induced changes in rheobase in AIC-NAc projection neurons. An unpaired *t*-test showed that heroin administration significantly increases rheobase relative to control treated animals (*t*_68_ = 3.3; *p* < 0.01; Figure 5A). Next, we examined heroin-induced changes in action potential characteristics. Unpaired *t*-tests showed no significant change in action potential amplitude (*t*_68_ = 0.7; *p* > 0.05; Figure 5B), rise time (*t*_68_ = 1.8; *p* > 0.05; Figure 5C), or decay time (*t*_68_ = 0.02; *p* > 0.05; Figure 5D). We next assessed changes in action potential output number induced by stepped depolarizing currents. Specifically, we assessed the number of action potentials elicited by varying amplitudes of injected currents. Example current injections and cellular responses can be seen in Figure 5E. A two-way ANOVA with treatment (heroin and saline) and input current amplitude as factors, revealed significant effects of current input (*F*_14,991_ = 25.2; *p* < 0.0001), treatment (*F*_1,991_ = 91.2; *p* < 0.0001) as well as a significant interaction between these two factors (*F*_14,991_ = 5.8; *p* < 0.0001). A Bonferroni’s *post hoc* comparison revealed a significant difference in the number of spikes elicited between saline and heroin treated animals at current inputs at and above 100pA. Heroin significantly decreased the number of action potentials elicited relative to saline, with depolarizing currents of 100 pA (*t*_991_ = 3.5; *p* < 0.01), 115 pA (*t*_991_ = 4.9; *p* < 0.0001), 130 pA (*t*_991_ = 5.0; *p* < 0.0001), 145 pA (*t*_991_ = 5.4; *p* < 0.0001), 160 pA (*t*_991_ = 5.8; *p* < 0.0001), and 175 pA (*t*_991_ = 6.0; *p* < 0.0001). These results are shown in Figure 5F.

## Discussion

In the present study, we explored the effects of LBN rearing conditions on heroin self-administration, progressive ratio responding, extinction and cue-induced reinstatement. Additionally, we examined the effects of repeated heroin or saline exposure on AIC-NAc projecting pyramidal cell electrophysiology and excitability in LBN and control reared animals. Here we report LBN has no significant effect, relative to control rearing, on the acquisition of heroin self-administration in either male or female animals. Due to the lack of sex differences in acquisition, data from female and males were collapsed within each group for subsequent analyses. When placed into a progressive ratio schedule of reinforcement, LBN reared animals earned significantly more infusions than control reared animals. LBN reared animals also showed impaired extinction learning relative to controls both before and after reinstatement testing, and LBN reared animals also showed potentiated cue-induced reinstatement of heroin-seeking. These observations are highly consistent with a recently published study that demonstrated LBN rearing conditions did not alter acquisition of heroin self-administration, but did impair extinction learning and potentiate cue-induced reinstatement (Levis et al., 2019). Taken together, these data suggest that while LBN does not necessarily affect the initiation of heroin use, it may prevent inhibition of drug seeking as well as potentiate relapse or responsivity to heroin-associated cues.

Furthermore, we demonstrate that LBN has no effect on baseline electrophysiological properties or excitability of AIC-NAc projecting pyramidal cells, which have recently been implicated in opioid reward and relapse (Zhang et al., 2019; Wang et al., 2021). However, we demonstrate that regardless of rearing condition, non-contingent heroin exposure over five continuous days significantly reduces the excitability of AIC-NAc projecting pyramidal cells by increasing rheobase and reducing the number of action potentials elicited by current injections. Taken together, these results demonstrate that LBN may not alter AIC-NAc connectivity initially, but continuous heroin exposure may have the ability to alter the excitability of this pathway, which could give rise to inappropriate functional connectivity resulting in behavioral phenotypes such as continued heroin seeking and increased vulnerability to relapse.

### The Effects of Early Life Stress on Heroin Use Behaviors

Heroin users have been found to experience higher levels of early life stress including childhood trauma such as physical punishment, physical neglect and penetrative sexual abuse (Grice et al., 1995; Moustafa et al., 2021). Exposure to ELS in rodents have validated such findings, demonstrating that ELS increases drug self-administration and relapse-related behaviors (Duffing et al., 2014; Koob and Schulkin, 2019). Furthermore, others have shown that maternal deprivation in rat pups increases the rewarding effects of morphine and promotes morphine seeking propensities and dependence in adulthood (Vazquez et al., 2005, 2006). For a more in depth review regarding ELS and the risks for opioid misuse, we direct the readers to the recent review by Oswald and colleagues (Oswald et al., 2021). Most similar to the study conducted here, the Mahler laboratory has also shown that LBN had no effect on overall heroin intake, yet promotes cue-induced reinstatement of heroin-seeking as well as impaired extinction learning in rats (Levis et al., 2019), which is consistent with the results we have reported here. In addition, we also demonstrated that LBN reared animals show higher breakpoints for heroin (increased number of heroin infusions earned) compared to control reared animals, demonstrating that these animals show elevated motivation for heroin. Taken together, our results suggest that LBN does not alter acquisition of heroin self-administration, yet increases motivation for the drug under conditions of increasing behavioral demand, reduces inhibitory learning with regards to heroin seeking (i.e., extinction) and increases the propensity for relapse (i.e., reinstatement) induced by heroin-associated cues.

### Changes in Anterior Insular Excitability Induced by Heroin

Previous studies have shown that insular structure and function are adversely affected by both opiates and ELS. In fact, a meta-analysis of 12 neuroimaging studies of opiate-dependent individuals has recently shown evidence for gray matter atrophy within the insular cortex, accompanied by abnormal functional connectivity, resting state activity as well as white matter composition (Wollman et al., 2017). Similarly, individuals exposed to ELS showed reduced cortical thickness and volume in the insula (Baker et al., 2013; McLaughlin et al., 2014; Mackey et al., 2017; Lim et al., 2018), and rodents subjected to maternal stress or early life social isolation show neuronal loss in the AIC (Poeggel et al., 2005). ELS and repeated opiate exposure also affects the morphology and physiology of neurons in efferent targets of the AIC, including the NAc (Aleksic et al., 2016; Thompson et al., 2021). In the current study, we show that ELS alone did not have an effect on basic cellular physiology or excitability of AIC-NAc projecting pyramidal neurons. However, continuous exposure to heroin for five consecutive days resulted in decreased excitability of AIC-NAc projection neurons. We and others have found similar effects within the prefrontal cortex, where heroin decreases the AMPA/NMDA ratios in layer V pyramidal neurons through reductions in AMPA receptor expression and activity (Van den Oever et al., 2008) and decreases layer V pyramidal cell excitability of commissural projecting pyramidal neurons (Leyrer-Jackson et al., 2021). Further, non-contingent morphine exposure has also been shown to reduce cellular excitability within the mPFC as reflected by reductions in activity-related immediate early gene c-fos expression (Piao et al., 2017). Furthermore, heroin has been shown to increase GABAergic inhibition in the mPFC, therefore reducing pyramidal cell excitability (Van den Oever et al., 2008). Taken together, these studies highlight that heroin exposure has the ability to decrease pyramidal cell excitability within the prefrontal cortex through mechanisms that may also be observed within the anterior insula.

### Limitations of the Current Study

In the current study, we did not examine electrophysiological changes in AIC-NAc pyramidal neurons following heroin self-administration, extinction or reinstatement. Given that experimenter delivered and IVSA can have different effects on cellular excitability, it is possible that AIC-NAc pyramidal neurons display differences following each of these phases of IVSA as compared to experimenter delivered used in the current study. While there are limited studies addressing the differences in AIC-NAc pathway following contingent versus non-contingent heroin delivery, studies assessing effects of heroin on other pathways have reported some physiological differences. For example, non-contingent heroin delivery was found to reduce GABAergic neuronal firing within the ventral tegmental area (VTA), whereas heroin self-administration (contingent) initially results in a burst of GABAergic activity during nose-poke/approach activity, which was followed by a dramatic reduction in firing, below pre-approach levels (Steffensen et al., 2006). As well, other studies utilizing contingent and non-contingent cocaine and 3,4-methyledoxymethamphetamine (MDMA) delivery have shown enhanced dopamine transporter binding within the accumbens and VTA (Miguéns et al., 2008), and reduced dopamine levels within the NAc (Orejarena et al., 2009) following contingent administration relative to non-contingent administration of each drug, respectively. Given that these studies demonstrate physiological changes that vary between contingent and non-contingent drug administration paradigms, assessment of self-administered heroin effects on AI-NAc projections is an important future avenue to pursue. However, such a paradigm would need to incorporate the degree of heroin intake and parse the effects of low vs. high drug intake to make definitive claims in regards to the results presented in the current study. Nonetheless, the electrophysiological experiments conducted here are an important first step in understanding how heroin exposure can alter this specific reward pathway.

In the current study, we did not observe differences between AIC-NAc projection neurons recorded from heroin-treated LBN and control reared animals. This finding suggests that heroin significantly reduces the excitability of this pathway, regardless of LBN exposure. However, these lack of effects are possibly due to the short duration (i.e., 5 days) of heroin exposure. Given that LBN vs. control animals showed no differences in heroin IVSA in Experiment 1, it is not surprising that experimenter delivered heroin did not produce any differences in AIC-NAc excitability between ELS and control reared animals. It is likely that longer exposure (15 + days as used in the IVSA paradigm) to heroin as well as withdrawal would reveal differences in AIC-NAc projection neuron excitability between ELS and control reared animals. To fully explore this hypothesis, future studies exploring the effects long-term heroin exposure as well as heroin withdrawal on this pathway are necessary. Additionally, these studies would lend insight into potential neuronal underpinnings that lead to hindered extinction to heroin-paired cues as well as

## Conclusion

In the current study, we demonstrated that LBN during early postnatal development increases the vulnerability toward heroin seeking behavior later in life, as manifested by impaired extinction learning and increased reinstatement magnitude in response to heroin-paired cues. To our knowledge, we have also demonstrated for the first time that LBN increases demand for heroin as assessed by progressive ratio procedures. Furthermore, we demonstrate that repeated non-contingent exposure to heroin has inhibitory effects on the excitability AIC-NAc projection neurons, which could significantly alter connectivity between these two regions and promote inappropriate plasticity within the accumbens. In agreement with this, it was recently demonstrated *via* functional connectivity analyses in heroin-dependent patients that degree centrality values for the insula and NAc were negatively correlated with relapse frequency (Wang et al., 2021). Taken together, these findings suggest that ELS promotes vulnerability to various aspects of OUDs and related behaviors. Further, these results provide evidence for heroin induced changes in AIC-NAc projection neuron excitability that may promote OUDs.

## Data Availability Statement

The raw data supporting the conclusions of this article will be made available by the authors, without undue reservation.

## Ethics Statement

The animal study was reviewed and approved by Institutional Animal Care and Use Committee, Arizona State University.

## Author Contributions

JL-J performed the electrophysiology experiments and analyses and wrote the original draft of the manuscript. PO and EN performed surgical procedures, conducted the behavioral experiments, and edited the manuscript. MFO conceptualized the study, performed surgical procedures, conducted analyses, and edited the manuscript. All authors contributed to the article and approved the submitted version.

## Conflict of Interest

The authors declare that the research was conducted in the absence of any commercial or financial relationships that could be construed as a potential conflict of interest.

## Publisher’s Note

All claims expressed in this article are solely those of the authors and do not necessarily represent those of their affiliated organizations, or those of the publisher, the editors and the reviewers. Any product that may be evaluated in this article, or claim that may be made by its manufacturer, is not guaranteed or endorsed by the publisher.
